# Weight bias, stigma and discrimination: a call for greater conceptual clarity

**DOI:** 10.3389/fpsyg.2025.1710851

**Published:** 2025-11-06

**Authors:** Marilou Côté, Vida Forouhar, Sabrina Sacco, Aurélie Baillot, Mary Himmelstein, Brad Hussey, Angela C. Incollingo Rodriguez, Taniya S. Nagpal, Sarah Nutter, Ian Patton, Rebecca L. Pearl, Rebecca M. Puhl, Ximena Ramos Salas, Shelly Russell-Mayhew, Angela S. Alberga

**Affiliations:** 1 Faculté des Sciences de l'éducation, Université Laval, Québec, QC, Canada; 2 Centre Nutrition, Santé et Société (NUTRISS), Institut sur la Nutrition et les Aliments Fonctionnels (INAF), Université Laval, Québec, QC, Canada; 3 Department of Health, Kinesiology, and Applied Physiology, Concordia University, Montreal, QC, Canada; 4 Department of Psychology, Concordia University, Montreal, QC, Canada; 5 École Interdisciplinaire de la Santé, Université du Québec en Outaouais, Gatineau, QC, Canada; 6 Department of Psychological Sciences, Kent State University, Kent, OH, United States; 7 Bias 180, Dundas, ON, Canada; 8 Replica Communications, Dundas, ON, Canada; 9 Psychological & Cognitive Sciences, Worcester Polytechnic Institute, Worcester, MA, United States; 10 Faculty of Kinesiology, Sport, and Recreation, University of Alberta, Edmonton, AB, Canada; 11 Department of Educational Psychology and Leadership Studies, University of Victoria, Victoria, BC, Canada; 12 Obesity Canada, Edmonton, AB, Canada; 13 Department of Clinical and Health Psychology, University of Florida, Gainesville, FL, United States; 14 Department of Human Development and Family Sciences, University of Connecticut, Storrs, CT, United States; 15 Replica Communications, Kristianstad, Sweden; 16 Werklund School of Education, University of Calgary, Calgary, AB, Canada

**Keywords:** weight bias, weight stigma, weight discrimination, definition, terms, concepts, operationalization

## Introduction

Weight bias, weight stigma, and weight discrimination are some of the terms that have been used interchangeably to represent the negative attitudes, stereotypes, and behaviors toward individuals based on their body size, weight, and/or shape. Despite the exponential increase in research in this field over the last 20 years and increased awareness of this social justice issue worldwide (Nutter et al., 2018), there is an inconsistent use of terms, definitions, and methods to define, differentiate, and measure weight bias, weight stigma, and weight discrimination concepts. Concepts such as bias, stigma, and discrimination are foundational in social psychology and other fields (Allport et al., 1954; Goffman, 1963). Despite this rich conceptual heritage, their application in weight stigma research remains inconsistent, with definitions often adapted, interpreted, or operationalized in ways that vary across studies and disciplines (e.g., psychology, medicine, public health, nursing, sociology).

In this opinion piece, we draw attention to specific challenges in the study of weight bias, stigma and discrimination related to the lack of clarity in usage of key terms and conceptual definitions, with the aim of encouraging future research that can inform efforts toward improving clarity. Rather than offering definitive solutions, this piece aims to highlight the need to increase: (1) conceptual clarity of terms; (2) scientific rigor of operationalization and measurement of these concepts, and (3) the generalizability and transferability of terminology used across languages and cultural contexts.

### Conceptual clarity and addressing the jingle-jangle fallacy

The “jingle fallacy” refers to using the *same term* to describe different phenomena (Thorndike, 1904; e.g., using the term “weight stigma” to describe different phenomena such as negative attitudes, negative emotions, and negative behaviors toward people with higher weights). The “jangle fallacy” refers to using *different terms* to describe the same phenomenon (Kelley, 1927; e.g., using the words weight stigma, weight bias, and weight discrimination interchangeably to describe the same phenomenon of holding negative attitudes toward people with higher weights). Publications showcase terms being used interchangeably, even though there may be important conceptual distinctions in how different researchers utilize different terms in the field of weight stigma research (Meadows and Higgs, 2020). For example, when authors use terms such as “weight bias,” “weight stigma,” and “weight discrimination” interchangeably, a reader may wonder whether study authors measured negative beliefs, attitudes, stereotypes, held by the sample population being studied or behaviors enacted toward others. In addition, various descriptors (e.g., enacted, experienced) are frequently combined with different terms (Table 1), potentially further complicating the reader's understanding of the research that was conducted and reported in the manuscripts (e.g., did the researchers measure negatives attitudes toward people with higher weights that were held by the sample population being studied? Or did the researchers measure the sample populations' own personal experiences of weight discrimination?). The jingle-jangle fallacy and inconsistencies in terminology could hinder progress in weight stigma research, knowledge translation, and advocacy by creating obstacles to clarity that contributes to misunderstanding of research results, and decreases the translation and application of research-generated knowledge with different end users (e.g., healthcare administrators, policy makers).

**Table 1:** Examples of descriptors and terms used in the weight stigma literature.

Descriptors	Anticipated; enacted; experienced; explicit; implicit; internalized; institutional; interpersonal; intrapersonal; perceived; social; structural etc.
Terms	Weight bias; weight discrimination; weight stigma; weight stigmatization; anti-fat attitude; anti-fat belief; anti-fat bias; anti-fat prejudice; body size stigma; fat phobia; sizeism; obesity stigma etc.

To improve our understanding of concepts used in the field of weight stigma research, it is first helpful to clarify the distinction among existing definitions of the terms used in the field of social psychology. The pioneering work of sociologist Goffman (1963) defined stigma as “an attribute that is deeply discrediting” (p. 3). Link and Phelan (2001) later argued that Goffman's definition did not comprehensively capture the nature of stigma and that there was great variability in the definitions of stigma used by different researchers. This could be because the concept of stigma has been applied in a wide range of situations and studied in an array of disciplines (e.g., sociology, psychology, public health, nursing, etc.). Accordingly, Link and Phelan concluded that, due to the complexity of stigma, it is advisable to allow variability in its definition, as long as researchers clearly specify what they mean by stigma when using the term (Link and Phelan, 2001). They defined stigma as a phenomenon that exists only if the five following components converge: (i) difference labeling (e.g., categorizing individuals as “thin” vs. “fat” based on their body weight); (ii) negative stereotypes (e.g., assuming people with higher weight are lazy and lack self-discipline); (iii) separating “us” from “them” (e.g., referring to individuals with higher weight as an outgroup); (iv) status loss and discrimination (e.g., differential treatment and inequities in employment or healthcare due to body size or weight); and (v) the dependence on power (e.g., the perpetuation of weight stigma in medical guidelines, which are controlled by influential organizations). In essence, their definition frames stigma as a social process of devaluation rooted in social norms and enacted through power structures, in which labeling, stereotyping, social exclusion, and status loss co-occur and are reinforced through cultural and institutional practices. However, within the field of weight stigma, the term “stigma” is often used inconsistently, sometimes referring only to certain aspects of this process (e.g., having negative stereotypes about people with higher weights). Rather than redefining these well-established concepts, the field would benefit from a more consistent and transparent application of existing definitions, thoughtfully adapted to the specificities of body weight as a socially devalued attribute. Clarifying how the terms related to weight stigma are understood and used—both conceptually and operationally—is essential to promote shared understanding and advance theoretical and empirical work in the field. This is especially relevant given that definitions of weight stigma often draw from a mix of foundational stigma literature (Goffman, 1963; Link and Phelan, 2001; Stuber et al., 2008) with some of the initial literature that emerged when the scientific and psychological study of weight stigma began to gain traction (e.g., Puhl and Brownell, 2001, 2003). Further conceptual work is needed to clarify how weight stigma is defined (including weight bias and weight discrimination, which are related terms), how terms are applied across studies and disciplines and how they may have evolved over time.

### Scientific rigor of operationalization and measurement

The conflation of concepts in weight stigma research can contribute to challenges in differentiating terms and therefore, it becomes challenging to determine what methods should be used to measure each of these concepts. At the 2015 National Weight Bias Summit in Canada, measurement of weight stigma was identified as a key research priority in the field (Alberga et al., 2016) and the most recent 2024 International Weight Bias Summit identified “Conceptual and Methodological Clarity” as one of the six themes that warrants future research in the field (Côté et al., under review)^1^. Recently, researchers in the field have raised questions about the validity and reliability of measures used to assess concepts, including internalized weight bias or stigma (e.g., Meadows and Higgs, 2020; Nutter et al., 2024; Pearl et al., 2023; Romano et al., 2022; Saunders et al., 2022), for example, where there is a lack of consistency in the definitions and terminology used (e.g., “weight self-stigma” vs. “internalized weight bias”), which have contributed to variability in the operationalization of this concept (Austen et al., 2021; Nutter et al., 2024). These inconsistencies create challenges in delineating internalized weight bias or stigma with other self-judgement concepts like self-esteem, body image, and body dissatisfaction (Meadows and Higgs, 2020; Austen et al., 2021). Previous work has revisited the conceptualization of internalized weight bias or stigma and found that existing measures, albeit psychometrically sound, may not entirely support the theoretical concept from which internalized weight bias or stigma has been constructed in the literature. For example, a recent study found that 45–66% of individuals with high scores on the most common measure of internalized weight stigma did not endorse negative weight stereotypes in a semi-structured interview (Pearl et al., 2023). Similarly, it has been suggested that the conceptualization of weight bias is not reflected in its operationalization, since the most popular measures for assessing weight bias often include items that are not consistent with widely used definitions of the term (e.g., causes of, consequences of, and solutions to obesity; Stewart and Ogden, 2021). These challenges could ultimately hinder researchers' abilities to accurately understand the implications of their results and contribute to misinterpretation of these concepts (Nutter et al., 2024).

Challenges with the conceptualization and operationalization of terms in the field of weight stigma could result in conflation of concepts, creating obstacles for evidence synthesis and knowledge translation. Building upon and refining existing measures of weight bias, weight stigma and weight discrimination can potentially improve the comparison of results between existing studies and the synthesis of existing evidence. Thus, future research should aim to provide guidance on aligning measurement tools more closely with underlying concepts, thereby supporting clearer messaging, more effective knowledge translation, and improved implementation of research findings.

### Generalizability and transferability to different languages, cultures, contexts, and settings

People experience weight stigma around the world (Puhl et al., 2021), which has prompted more research internationally, including countries where English is not the official language (Brewis et al., 2018; Eggerichs et al., 2023). International research on weight stigma is important and can guide our understanding of linguistic and cultural impacts of negative weight-related social norms, perceptions, attitudes, beliefs, and behaviors. Such studies can also contribute to assessing global trends in weight stigmatizing attitudes and experiences.

International studies have also reported weight stigma in many different settings including in interpersonal relationships (Lawrence et al., 2023), community settings (Puhl et al., 2021), healthcare (Ryan et al., 2023), education (Nutter et al., 2019), employment (Giel et al., 2010) and in the media (Kite et al., 2022). By measuring global trends and understanding the nature of negative attitudes, beliefs, and behaviors about body weight across countries, regions, cultures, and settings, we can also develop interventions that are more effective as well as linguistically and culturally appropriate and sensitive.

Thus, gaining a better understanding of the international use of terms in different countries and understanding their translation and application across languages, cultures, and settings is crucial for ensuring appropriate linguistic adaptations and validation studies, improving the generalizability of findings, enabling cross-cultural comparisons, and highlighting research priorities that reflect weight stigma concepts globally. These efforts could also foster more effective international collaborations, increase transparency of ongoing research, reduce redundancy in research, and assist with the accumulation and synthesis of evidence.

## Discussion

This opinion piece highlights three key challenges in the conceptualization and application of weight bias, weight stigma, and weight discrimination. First, conceptual clarity is needed to address jingle-jangle fallacies and ensure consistent use of terms such as weight stigma, weight bias, and weight discrimination. Second, improving the rigor of operationalization and measurement is essential to accurately capture these concepts across studies. Third, enhancing the generalizability and transferability of findings across languages, cultures, contexts, and settings requires understanding how terminology is used internationally and working toward greater alignment in its translation and application. Together, these challenges underscore the need for future research aimed at clarifying usage, measures, and applications, facilitating cross-cultural comparability, and supporting coordinated international efforts in the field.

## Footnotes

1 Côté, M., Forouhar, V., Sacco, S., González-González, M., Baillot, A., Himmelstein, M., et al. (under review). Results of the international weight bias summit: establishing future research directions in the field.

## References

[B1] Alberga A. S. Russell-Mayhew S. von Ranson K. M. McLaren L. Ramos Salas X. Sharma A. M. (2016). Future research in weight bias: what next? Obesity 24, 1207–1209. doi: 10.1002/oby.21480 27129601

[B2] Allport G. W. Clark K. Pettigrew T. F. (1954). The nature of prejudice. Addison-Wesley.

[B3] Austen E. Pearl R. L. Griffiths S. (2021). Inconsistencies in the conceptualisation and operationalisation of internalized weight stigma: a potential way forward. Body Image 36, iii–v. doi: 10.1016/j.bodyim.2020.12.002 33358360 PMC8500548

[B4] Brewis A. SturtzSreetharan C. Wutich A. (2018). Obesity stigma as a globalizing health challenge. Globaliz. Health 14, 1–6. doi: 10.1186/s12992-018-0337-x 29439728 PMC5811962

[B5] Eggerichs L. A. Wilson O. W. A. Chaplin J. E. Ramos Salas X. (2023). Weight stigma in Latin America, Asia, Middle East, and Africa: a scoping review. Obesity Facts. 17, 217–226. doi: 10.1159/000536554 PMC11149978 38316119

[B6] Giel K. E. Thiel A. Teufel M. Mayer J. Zipfel S. (2010). Weight bias in work settings – a qualitative review. Obes. Facts 3, 33–40. doi: 10.1159/000276992 20215793 PMC6452122

[B7] Goffman E. (1963). Stigma: Notes on the Management of Spoiled Identity. New York, NY: Simon and Sc.

[B8] Kelley T. L. (1927). Interpretation of Educational Measurements. San Diego, CA: World Book Co.

[B9] Kite J. Huang B. H. Laird Y. Grunseit A. McGill B. Williams K. . (2022). Influence and effects of weight stigmatisation in media: a systematic review. eClinicalMedicine 48, 1–11. doi: 10.1016/j.eclinm.2022.101464 PMC9125650 35706492

[B10] Lawrence S. E. Puhl R. M. Watson R. J. Schwartz M. B. Lessard L. M. Foster G. D. (2023). Family-based weight stigma and psychosocial health: a multinational comparison. Obesity 31, 1666–1677. doi: 10.1002/oby.23748 37171908

[B11] Link B. G. Phelan J. C. (2001). Conceptualizing stigma. Annu. Rev. Sociol. 27, 363–385. doi: 10.1146/annurev.soc.27.1.363

[B12] Meadows A. Higgs S. (2020). A bifactor analysis of the Weight Bias Internalization Scale: what are we really measuring? Body Image 33, 137–151. doi: 10.1016/j.bodyim.2020.02.013 32155463

[B13] Nutter S. Ireland A. Alberga A. S. Brun I. Lefebvre D. Hayden K. A. . (2019). Weight bias in educational settings: a systematic review. Curr. Obes. Rep. 8, 185–200. doi: 10.1007/s13679-019-00330-8 30820842

[B14] Nutter S. Russell-Mayhew S. Arthur N. Ellard J. H. (2018). Weight bias as a social justice issue: a call for dialogue. Can. Psychol. 59, 89–99. doi: 10.1037/cap0000125

[B15] Nutter S. Saunders J. F. Waugh R. (2024). Current trends and future directions in internalized weight stigma research : a scoping review and synthesis of the literature. J. Eat. Disord. 12, 1–15. doi: 10.1186/s40337-024-01058-0 39010124 PMC11247756

[B16] Pearl R. L. Wadden T. A. Groshon L. C. Fitterman-Harris H. F. Bach C. LaFata E. M. (2023). Refining the conceptualization and assessment of internalized weight stigma: a mixed methods approach. Body Image 44, 93–102. doi: 10.1016/j.bodyim.2022.12.002 36549092

[B17] Puhl R. Brownell K. D. (2001). Bias, discrimination, and obesity. Obes. Res. 9, 788–805. doi: 10.1038/oby.2001.108 11743063

[B18] Puhl R. Brownell K. D. (2003). Ways of coping with obesity stigma: review and conceptual analysis. Eat. Behav. 4, 53–78. doi: 10.1016/S1471-0153(02)00096-X 15000988

[B19] Puhl R. M. Lessard L. M. Pearl R. L. Himmelstein M. S. Foster G. D. (2021). International comparisons of weight stigma: addressing a void in the field. Int. J. Obes. 45, 1976–1985. doi: 10.1038/s41366-021-00860-z 34059785

[B20] Romano K. A. Heron K. E. Sandoval C. M. Howard L. M. MacIntyre R. I. Mason T. B. (2022). A meta-analysis of associations between weight bias internalization and conceptually-related correlates: a step towards improving construct validity. Clin. Psychol. Rev. 92:102127. doi: 10.1016/j.cpr.2022.102127 35074712 PMC8858873

[B21] Ryan L. Coyne R. Heary C. Birney S. Crotty M. Dunne R. . (2023). Weight stigma experienced by patients with obesity in healthcare settings: a qualitative evidence synthesis. Obe. Rev. 24:e13606. doi: 10.1111/obr.13606 37533183

[B22] Saunders J. F. Nutter S. Russell-Mayhew S. (2022). Examining the conceptual and measurement overlap of body dissatisfaction and internalized weight stigma in predominantly female samples: a meta-analysis and measurement refinement study. Front. Glob. Women's Health 3, 1–16. doi: 10.3389/fgwh.2022.877554 35528312 PMC9070483

[B23] Stewart S. J. F. Ogden J. (2021). What are weight bias measures measuring? An evaluation of core measures of weight bias and weight bias internalisation. Health Psychol. Open 8, 1–10. doi: 10.1177/20551029211029149 34377524 PMC8323429

[B24] Stuber J. Meyer I. Link B. (2008). Stigma, prejudice, discrimination and health. Soc. Sci. Med. 67, 351–357. doi: 10.1016/j.socscimed.2008.03.023 18440687 PMC4006697

[B25] Thorndike E. L. (1904). An Introduction to the Theory of Mental and Social Measurements. New York, NY: The Science Press. doi: 10.1037/13283-000

